# The Role of Chloride in Cardiorenal Syndrome: A Practical Review

**DOI:** 10.3390/jcm14155230

**Published:** 2025-07-24

**Authors:** Georgios Aletras, Maria Bachlitzanaki, Maria Stratinaki, Ioannis Petrakis, Theodora Georgopoulou, Yannis Pantazis, Emmanuel Foukarakis, Michael Hamilos, Kostas Stylianou

**Affiliations:** 1 Department of Cardiology, Venizelio General Hospital of Heraklion, 71409 Heraklion, Greece or medp2012222@med.uoc.gr (G.A.); maria.stratinaki@gmail.com (M.S.); or medp2012134@med.uoc.gr (T.G.); mfouk@hotmail.com (E.F.); 2 School of Medicine, University of Crete, 70013 Heraklion, Greece or medp2011922@med.uoc.gr (M.B.); or petrakgia@uoc.gr (I.P.); or mchamilos@uoc.gr (M.H.); 3 Second Department of Internal Medicine, Venizelio General Hospital of Heraklion, 71409 Heraklion, Greece; 4 Department of Nephrology, University General Hospital of Heraklion, 71500 Heraklion, Greece; 5 Institution of Applied and Computational Mathematics, Foundation of Research and Technology-Hellas, 70013 Heraklion, Greece; pantazis@iacm.forth.gr; 6 Department of Cardiology, University General Hospital of Heraklion, 71500 Heraklion, Greece

**Keywords:** chloride, heart failure, cardiorenal syndrome, hypochloremia, hyperchloremia, diuretic resistance, neurohormonal activation

## Abstract

Chloride, long considered a passive extracellular anion, has emerged as a key determinant in the pathophysiology and management of heart failure (HF) and cardiorenal syndrome. In contrast to sodium, which primarily reflects water balance and vasopressin activity, chloride exerts broader effects on neurohormonal activation, acid–base regulation, renal tubular function, and diuretic responsiveness. Its interaction with With-no-Lysine (WNK) kinases and chloride-sensitive transporters underscores its pivotal role in electrolyte and volume homeostasis. Hypochloremia, frequently observed in HF patients treated with loop diuretics, is independently associated with adverse outcomes, diuretic resistance, and arrhythmic risk. Conversely, hyperchloremia—often iatrogenic—may contribute to renal vasoconstriction and hyperchloremic metabolic acidosis. Experimental data also implicate chloride dysregulation in myocardial electrical disturbances and an increased risk of sudden cardiac death. Despite mounting evidence of its clinical importance, serum chloride remains underappreciated in contemporary risk assessment models and treatment algorithms. This review synthesizes emerging evidence on chloride’s role in HF, explores its diagnostic and therapeutic implications, and advocates for its integration into individualized care strategies. Future studies should aim to prospectively validate these associations, evaluate chloride-guided therapeutic interventions, and assess whether incorporating chloride into prognostic models can improve risk stratification and outcomes in patients with heart failure and cardiorenal syndrome.

## 1. Introduction

Cardiorenal syndrome (CRS) encompasses a spectrum of disorders characterized by concurrent dysfunction in the heart and kidneys, where impairment in one organ can lead to acute or chronic impairment in the other [1]. Among these subtypes, acute CRS—defined as acute worsening of cardiac function leading to acute kidney injury—is the most common clinical presentation and remains a clinical challenge [2,3]. While a sodium-centric view has traditionally dominated the management of acute heart failure (HF) and cardiorenal syndrome, with dietary modifications primarily focused on lower sodium intake to avoid extracellular fluid volume expansion, emerging evidence challenges this conventional approach [4]. Some studies suggest that higher salt intake may not always lead to adverse consequences, and excessively low intake may paradoxically result in negative outcomes [5]. Some investigators have even explored the use of hypertonic saline solutions in treating acute HF [6,7].

This escalating controversy has prompted calls for a more cautious approach to rigorous sodium restriction in symptomatic HF patients [4]. This shift in perspective is perplexing, considering the well-established negative impact of increased total body sodium on renal, cardiac, and vascular homeostasis. However, some researchers have begun to shift their focus to chloride, the often-overlooked counter-ion of sodium in salt [8,9].

Chloride and sodium are both major potent ions in the extracellular fluid. While numerous studies have linked serum sodium abnormalities to poorer outcomes in acute or chronic HF, relatively few have accounted for chloride levels in their analyses [4]. Recent research suggests that chloride may be a stronger predictor of outcomes than sodium in HF [10,11,12,13], and the existing debate regarding the benefits and risks of salt restriction may be partly related to its effects on chloride homeostasis [14]. Changes in plasma volume, vasopressin secretion, and the renin–angiotensin–aldosterone system in worsening HF may be primarily mediated by serum chloride, rather than serum sodium levels [15]. Several studies have also reported an inverse association between serum chloride and mortality in patients with worsening HF, independent of serum sodium levels, suggesting its potential as a prognostic marker [14].

Having established the emerging importance of chloride in heart failure and cardiorenal syndrome, it is crucial to delve into the underlying mechanisms by which this anion influences these conditions. Unlike the relatively well-defined role of sodium in fluid balance and blood pressure regulation, the specific pathophysiological contributions of chloride are only beginning to be elucidated [4].

While several reviews have previously examined chloride physiology in heart failure and cardiorenal syndrome, this article aims to offer a more clinically oriented perspective. Beyond summarizing key mechanisms, it emphasizes the practical application of these insights—especially in the assessment and management of chloride disturbances.

We also highlight the emerging role of urine chloride as a practical biomarker for evaluating volume status and predicting diuretic response—an area that has received relatively little attention in earlier reviews. Furthermore, we review recent clinical trial data and present actionable strategies, including chloride repletion, acetazolamide use, and diuretic regimen optimization.

With this approach, the review seeks to bridge the gap between pathophysiological understanding and clinical implementation, offering clinicians a pragmatic guide while also identifying critical areas for future investigation.

## 2. Literature Research Strategy

To support this narrative review, a comprehensive literature search was conducted using PubMed (National Library of Medicine, Bethesda, MD, USA), Scopus (Elsevier, Amsterdam, The Netherlands), and Google Scholar (Google LLC, Mountain View, CA USA) up to June 2025. The search included English-language articles without date restrictions. The following key terms and combinations were used:

▪ “chloride” AND “heart failure”;
▪ “chloride” AND “cardiorenal syndrome”;
▪ “hypochloremia” OR “hyperchloremia” AND “prognosis”;
▪ “diuretic resistance” AND “chloride”;
▪ “WNK kinases” AND “chloride”;
▪ “loop diuretics” AND “electrolyte balance”;
▪ “chloride” AND “neurohormonal activation”;
▪ “chloride channels” AND “arrhythmias”.

Keywords were selected based on their relevance to the key pathophysiological mechanisms and clinical outcomes associated with chloride disturbances in heart failure and cardiorenal syndrome. Additional references were identified by manually screening bibliographies of relevant articles and recent reviews. Priority was given to original research articles, large cohort studies, meta-analyses, and clinical trials.

Reference management was performed using Zotero version 6.0 (Center for History and New Media, George Mason University, Fairfax, VA, USA).

## 3. Renal Chloride Handling and Clinical Implications in Heart Failure

Chloride is the principal anion in the extracellular fluid and plays a central role in maintaining acid–base balance, osmotic pressure, and overall electrolyte homeostasis. It contributes approximately one-third of extracellular tonicity and works in close conjunction with sodium to regulate serum osmolarity and fluid distribution. Importantly, chloride maintains electroneutrality by balancing the positive charges of extracellular cations. Its inverse relationship with serum bicarbonate is fundamental to the body’s buffering system, which preserves physiologic pH. As such, fluctuations in chloride levels can significantly impact acid–base status [4,16].

**Figure 1:**
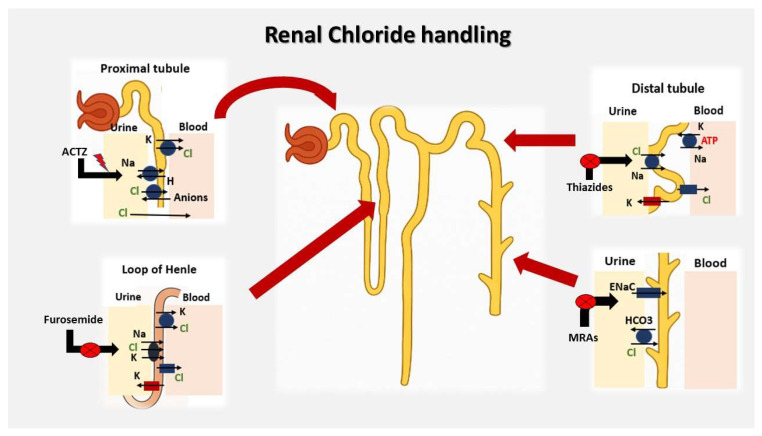
Renal chloride handling: The majority of filtered chloride is reabsorbed in the proximal tubule via both passive and active transcellular mechanisms involving Cl^−^/base exchangers and Na^+^/H^+^ exchangers. Acetazolamide (ACTZ), by inhibiting carbonic anhydrase, indirectly promotes proximal chloride reabsorption. In the thick ascending limb of the loop of Henle, chloride is reabsorbed through the Na^+^/K^+^/2Cl^−^ cotransporter (NKCC2), the pharmacologic target of loop diuretics such as furosemide. In the distal convoluted tubule, thiazide-sensitive Na^+^/Cl^−^ cotransporters and Cl^−^/HCO_3^−^_ exchangers mediate further reabsorption. The collecting duct finalizes chloride handling via pendrin and sodium-dependent Cl^−^/HCO_3^−^_ exchangers in intercalated cells. Sites of action for furosemide, thiazides, acetazolamide (ACTZ), and mineralocorticoid receptor antagonists (MRAs) are highlighted. Abbreviations: ACTZ: acetazolamide; ATP: Adenosine Triphosphate; ENaC: epithelial sodium channel; MRA: mineralocorticoid receptor antagonist; NKCC2: Na^+^/K^+^/2Cl^−^ cotransporter. This figure is an original illustration created by the authors for educational purposes.

Chloride is primarily handled by the kidneys, which work alongside the gastrointestinal tract to maintain its balance in the body. Most of the filtered chloride is reabsorbed in the proximal tubule—about 60%—through both passive pathways between cells and active transport mechanisms. Passive reabsorption occurs down the electrochemical gradient through tight junctions, while active transcellular chloride transport is mediated primarily by Cl^−^/base exchangers, working in concert with Na^+^/H^+^ exchangers (NHEs). Acetazolamide (ACTZ), a carbonic anhydrase inhibitor, indirectly promotes chloride retention by inhibiting proximal tubular sodium and bicarbonate reabsorption. The resulting accumulation of bicarbonate in the tubular lumen increases the negative electrochemical gradient, thereby enhancing chloride reabsorption to maintain electroneutrality and raising serum chloride levels [14,17]. Further chloride reabsorption occurs in the thick ascending limb of the loop of Henle via the Na^+^/K^+^/2Cl^−^ cotransporter (NKCC2), which is the pharmacologic target of loop diuretics. In the distal convoluted tubule, around 5% of chloride is reabsorbed through the thiazide-sensitive Na^+^/Cl^−^ cotransporter and Cl^−^/HCO_3^−^_ exchangers. The collecting duct finalizes chloride handling through reabsorption by intercalated cells via pendrin (a sodium-independent Cl^−^/HCO_3^−^_ exchanger) and sodium-dependent chloride–bicarbonate exchangers [14,16] (Figure 1).

Disruptions in chloride homeostasis, particularly hypochloremia, are common in heart failure and have been associated with worse clinical outcomes, regardless of the underlying left ventricular ejection fraction [18,19]. Hypochloremia is common in this setting, often due to aggressive diuresis or shifts in fluid status. It has been linked to reduced glomerular filtration rate (GFR) and worse outcomes, including higher risk of mortality and acute kidney injury. In contrast, hyperchloremia, often resulting from chloride-rich intravenous fluids, may provoke renal vasoconstriction through tubuloglomerular feedback and increase metabolic demands at the cellular level [14].

Notably, patients with low chloride levels often fall into two distinct profiles. One group has both low chloride and low sodium, typically due to fluid overload and dilution. The other has low chloride with normal sodium, suggesting true depletion—often from diuretics—and is frequently accompanied by elevated bicarbonate and low potassium levels. This second group may be especially prone to diuretic resistance and arrhythmias, highlighting the importance of recognizing these biochemical patterns to guide treatment [16].

## 4. The Importance of Chloride in Cardiorenal Syndrome

### 4.1. Hypochloremia

As previously noted, hypochloremia—often defined as a serum chloride concentration of less than 96 mmol/L—typically presents with two distinct biochemical profiles: one associated with water excess and hemodilution, and the other reflecting true chloride depletion [15,16].

The first mechanism underlying hypochloremia is dilution, or water excess, resulting from the non-osmotic release of arginine vasopressin. In the setting of decreased effective circulating volume, baroreceptor activation stimulates vasopressin secretion, promoting free water reabsorption in the collecting ducts independently of sodium and chloride. This leads to dilution of serum electrolytes and contributes to the development of hyponatremia and hypochloremia [20,21,22]. This pathophysiologic mechanism is supported by studies in patients with heart failure demonstrating increased circulating vasopressin levels during periods of decompensation [23].

However, hypochloremia only partially overlaps with hyponatremia; therefore, the second etiology of hypochloremia is chloride depletion. This phenomenon is frequently linked to the use of loop diuretics, which are a cornerstone of heart failure management and promote a disproportionately greater loss of chloride relative to sodium [14]. Notably, differences among loop diuretics have been observed; for instance, bumetanide has been associated with a more pronounced chloruretic effect compared to furosemide, despite producing similar levels of sodium and potassium excretion [24].

Recent evidence has established hypochloremia as an independent predictor of adverse cardiovascular outcomes, both in the general population and among patients with heart failure. Importantly, this association persists even after adjusting for traditional cardiovascular risk factors and other electrolytes, including serum sodium. Emerging studies suggest that serum chloride may serve as a more powerful prognostic marker than sodium, particularly in chronic heart failure [14]. A post hoc analysis of the Beta-Blocker Evaluation of Survival Trial (BEST) demonstrated that both hypochloremia and hyponatremia were linked to increased mortality at baseline, three months, and twelve months; however, after multivariable adjustment, only hypochloremia remained significantly associated with mortality risk [12]. Similarly, Grodin et al. [18] reported that, in a cohort of stable heart failure patients undergoing elective coronary angiography, each standard deviation decrease in serum chloride (approximately 4.1 mEq/L) at admission was associated with a 29% higher risk of five-year mortality, independent of serum sodium levels, medication use, cardiorenal biomarkers, and functional status [11]. Further reinforcing these findings, analysis of the TOPCAT trial revealed that lower serum chloride concentrations were correlated with greater risks of heart failure hospitalization, cardiovascular death, and all-cause mortality [18].

Although numerous studies have reported an association between hypochloremia and adverse outcomes in both acute and chronic heart failure, the causal relationship remains uncertain. These associations are frequently influenced by heterogeneous patient populations, variable comorbidities, and residual confounding factors. While observational data are consistent and abundant, interventional trials, specifically targeting serum chloride, have yielded inconclusive results. Consequently, it remains unclear whether hypochloremia plays a direct pathogenic role or merely reflects greater disease severity and worse prognosis [11,12,18,25].

Initial hypochloremia has been associated with a significantly higher incidence of hyponatremia after a 3-month follow-up [26]. The “chloride theory” of heart failure posits that chloride plays a more significant role in heart failure prognosis, neurohumoral activation, and diuretic resistance than previously recognized [14,27]. Hypochloremia is also related to diuretic resistance in heart failure [16], which will be discussed in the following sections.

### 4.2. Hyperchloremia

While hypochloremia has received considerable attention in the context of heart failure (HF), recent studies suggest that both low and high serum chloride levels may be associated with adverse clinical outcomes, indicating a possible U-shaped relationship [12,15,18]. Hyperchloremia, typically defined as a serum chloride concentration exceeding 105–115 mmol/L—though thresholds may vary across laboratories—has emerged as a potential contributor to poor prognosis in this population [15].

Hyperchloremia can result from various mechanisms, most notably excessive chloride administration through intravenous saline solutions or significant electrolyte-free water loss, such as in cases of gastrointestinal fluid depletion [15]. Importantly, hyperchloremia may also manifest independently or as part of a broader acid–base disturbance, namely hyperchloremic metabolic acidosis. This form of metabolic acidosis is characterized by an elevated chloride level coupled with a reduction in serum bicarbonate and a concomitant drop in blood pH. Understanding the underlying etiology is essential for proper management and may involve both renal and extrarenal origins [15,16].

Renal causes of hyperchloremic metabolic acidosis include both proximal and distal renal tubular acidosis (RTA). In proximal RTA, impaired reabsorption of bicarbonate by the renal tubules leads to bicarbonate wasting and compensatory chloride retention to maintain electrical neutrality and plasma volume. In distal RTA, the kidneys fail to adequately regenerate bicarbonate, impairing acid buffering and further promoting a hyperchloremic state. In both cases, the imbalance contributes to systemic acidosis, which can adversely affect cardiovascular function [28].

Extrarenal causes, such as secretory diarrhea, represent another common source of hyperchloremic metabolic acidosis. In this setting, bicarbonate is actively secreted into the intestinal lumen and lost in stool, while chloride is passively reabsorbed. The resulting electrolyte imbalance can significantly disrupt systemic acid–base status and plasma volume—effects that are particularly deleterious in heart failure patients due to their impaired renal compensatory capacity, neurohormonal activation, and heightened sensitivity to volume depletion [20,29].

Although the clinical consequences of hyperchloremia in HF are less well studied than those of hypochloremia, the existing literature suggests that elevated serum chloride may be associated with increased morbidity and mortality. Whether this association is causal or reflects underlying comorbidities and treatment strategies—such as high-volume chloride-rich fluid administration—remains unclear. Nevertheless, these findings underscore the need for a more nuanced approach to electrolyte monitoring and fluid management in patients with heart failure [15].

Despite these emerging associations, the body of evidence on hyperchloremia in heart failure remains limited compared to hypochloremia. Most available data are derived from retrospective analyses or broader electrolyte studies, rather than trials specifically designed to investigate elevated chloride levels in heart failure. Additionally, variability in the definition of hyperchloremia and confounding factors—such as fluid administration practices—further complicate interpretation. As a result, while the pathophysiological rationale is compelling, the clinical relevance of hyperchloremia in HF remains underexplored and warrants further prospective investigation [11,15,25].

In conclusion, hyperchloremia—particularly when associated with metabolic acidosis—may carry important implications for prognosis and management in heart failure. Its role as a marker or mediator of adverse outcomes deserves more focused research, and clinicians should remain vigilant when correcting fluid and electrolyte imbalances, especially in acutely decompensated patients [15].

### 4.3. Chloride and Neurohormonal Activation

In the setting of heart failure, where impaired cardiac function or altered ventricular filling compromises tissue perfusion, the activation of neurohormonal systems, such as the renin–angiotensin–aldosterone system and the sympathetic nervous system, represents an attempt to restore circulatory stability. While this response is initially compensatory, its prolonged activation promotes fluid retention, vasoconstriction, and myocardial remodeling, all of which are hallmarks of heart failure progression [14,20,21]. Traditionally, sodium has been considered the primary electrolyte driving these compensatory mechanisms. However, emerging evidence now highlights the critical regulatory role of chloride, which exhibits distinct physiological functions [8].

Chloride exerts a direct influence on renin secretion via its interaction with the macula densa, an essential sensor within the nephron. Reduced chloride delivery to this region—common in hypochloremic states—triggers enhanced renin release, independent of sodium concentration [14]. This phenomenon underscores chloride’s unique role in tubuloglomerular feedback, with animal studies demonstrating that chloride supplementation alone, not sodium, can suppress renin in sodium-restricted models [30,31]. These findings have been mirrored in clinical observations, particularly among HF patients on loop diuretics, where lower serum chloride is associated with elevated renin levels even after adjusting for serum sodium [23].

Beyond renin–angiotensin–aldosterone system (RAAS) activation, chloride deficiency contributes to heightened sympathetic outflow, particularly affecting renal hemodynamics. This results in afferent arteriolar constriction, impaired renal perfusion, and diminished natriuresis and diuresis—factors that potentiate fluid overload.

The so-called “chloride theory” proposes that serum chloride fluctuations are not merely secondary to volume changes but may actively influence plasma volume, RAAS activity, and vasopressin regulation [32]. For instance, alterations in serum chloride may activate central thirst pathways and disrupt vasopressin release, thereby contributing to volume dysregulation. A decrease in serum chloride, as commonly seen with loop diuretic use in heart failure, is thought to stimulate RAAS activation, enhance vasopressin release, and trigger central thirst mechanisms, all of which contribute to sodium and water retention. Conversely, restoration or elevation of serum chloride has been associated with renin suppression and improved diuretic responsiveness, suggesting that chloride itself plays a regulatory role in fluid homeostasis beyond its role as a passive anion [15]. Moreover, clinical studies suggest a prognostic significance of chloride levels in HF. In the VICTORIA trial, incremental increases in serum chloride were associated with reduced risk of cardiovascular mortality and HF hospitalizations, independent of natriuretic peptide levels [19]. Additionally, although both chloride and BNP have been associated with clinical outcomes, their interaction remains an area for further exploration [11].

In conclusion, chloride is now recognized as more than a passive electrolyte. Its role in neurohormonal modulation—particularly in RAAS and sympathetic activation—places it at the forefront of emerging strategies in HF management. Understanding and addressing chloride disturbances may provide an overlooked yet promising avenue to improve prognosis in patients with heart failure [15].

### 4.4. Chloride and Diuretic Resistance

Diuretic resistance represents a frequent and clinically significant barrier to effective decongestion in patients with heart failure (HF), particularly in the setting of chronic loop diuretic therapy. It is broadly characterized by an attenuated natriuretic response despite adequate dosing of diuretic agents and is associated with worse symptom burden, longer hospitalizations, and increased morbidity [33]. While traditionally attributed to impaired drug delivery or distal tubular adaptation, growing evidence highlights a key role for serum chloride concentration in modulating diuretic efficacy [14].

Hypochloremia is increasingly recognized as an independent predictor of poor diuretic response, even when controlling for serum sodium and bicarbonate levels. Low serum chloride correlates with reduced natriuresis per doubling of loop diuretic dose—a phenomenon known as impaired diuretic efficiency [23,34,35]. This relationship has been validated in multiple clinical cohorts, including a post hoc analysis of the ROSE trial, where lower baseline chloride levels were strongly associated with reduced responsiveness to loop diuretics and worse diuretic efficiency [36].

At the mechanistic level, chloride interacts with the With-no-Lysine K (WNK) family of kinases, which serve as intracellular sensors of chloride concentration and play a pivotal role in the regulation of renal sodium transport. These kinases modulate sodium–chloride transporters along the nephron, which include the Na-K-2Cl cotransporter in the thick ascending limb and the Na-Cl symporter in the distal convoluted tubule—both of which are targets of loop and thiazide diuretics. When intracellular chloride is low, WNK kinase activity increases, enhancing sodium and chloride reabsorption and counteracting the intended natriuretic effects [15]. Structural and biochemical studies have shown that chloride directly binds to the catalytic sites of WNK 1, KS-WNK 1, WNK 3, and WNK 4, stabilizing its inactive conformation; chloride depletion thus relieves this inhibition, triggering downstream activation [14,16,37,38].

While this upstream reabsorption does not directly cause potassium loss, it influences distal sodium delivery and aldosterone sensitivity in the collecting duct. In this setting, aldosterone promotes potassium excretion—especially when RAAS is activated—through a mechanism known as the “aldosterone paradox”, in which the kidney prioritizes either sodium retention or potassium loss depending on the dominant stimulus. As a result, chloride deficiency may indirectly contribute to hypokalemia and associated arrhythmias, particularly in heart failure patients treated with loop diuretics [8,15,37,38].

Furthermore, loop diuretics themselves can exacerbate hypochloremia, establishing a self-reinforcing loop of worsening electrolyte imbalance and decreasing therapeutic efficacy. Chronic loop diuretic exposure has also been shown to induce hypertrophic remodeling of the distal nephron, resulting in increased sodium reabsorption capacity downstream of the primary site of action [39]. These adaptations are particularly relevant in the setting of persistent hypochloremia and sustained WNK activity [14].

In addition to serum chloride, emerging evidence suggests that urine chloride (uCl^−^) may offer superior predictive value for identifying diuretic resistance in acute heart failure (AHF). In a prospective study of 50 patients undergoing standardized furosemide therapy, uCl^−^ concentrations ≥ 72 mmol/L were more closely associated with poor diuretic response than urine sodium (uNa^+^) levels, particularly when adjusted for urine creatinine. While the small sample size limits broad generalization, the findings support uCl^−^ as a promising biomarker for evaluating natriuretic efficiency and tubular responsiveness. Unlike uNa^+^, uCl^−^ appears less variable across post-diuretic timepoints, potentially offering a more stable and physiologically relevant marker in the setting of loop diuretic therapy [40]. Metabolic alkalosis, a common acid–base disturbance in decompensated HF, may further contribute to diuretic resistance in the context of chloride depletion. This form of alkalosis—historically referred to as “contraction alkalosis”—is now better conceptualized as “chloride-depletion alkalosis”. In the absence of adequate luminal chloride, pendrin-mediated Cl/HCO_3_ exchange in the collecting duct is impaired, perpetuating alkalemia and reducing natriuretic responsiveness [14].

Therapeutic strategies to address chloride-mediated diuretic resistance include chloride repletion via intravenous chloride-rich fluids or dietary intervention, combination diuretic therapy targeting different nephron segments, and consideration of alternative agents that bypass chloride-sensitive pathways, as discussed below [15,16]. Notably, increasing loop diuretic doses—a common clinical approach—may worsen hypochloremia and exacerbate resistance, emphasizing the need for more targeted interventions [14].

### 4.5. Chloride and Sudden Cardiac Death

Chloride plays a crucial but often underappreciated role in the electrical properties of myocardial cells, particularly in patients with heart failure (HF). Cardiac chloride channels contribute to ventricular repolarization and regulate the chronotropic activity of pacemaker cells, with additional involvement in maintaining myocyte volume and intracellular pH. Disruptions in chloride transport can impair membrane stability, prolong action potential duration, and promote excitation–contraction abnormalities, increasing susceptibility to arrhythmias and sudden cardiac death [15,16,41,42].

Experimental studies have shown that alterations in cardiac chloride channel activity, including in the sinoatrial node, directly affect membrane potentials and pacing function. Abnormal chloride levels can destabilize repolarization, partly through dysregulated intracellular pH and potassium homeostasis, both recognized arrhythmogenic factors [41,42]. Moreover, patients with HF demonstrate a significant reduction—approximately 50%—in the expression of the cystic fibrosis transmembrane conductance regulator (CFTR), an important chloride channel, during disease progression. This adaptive remodeling further contributes to electrical instability, arrhythmogenesis, and the structural changes that underlie myocardial hypertrophy and worsening heart failure [16].

Taken together, these findings extend the clinical relevance of chloride beyond fluid balance and neurohormonal regulation, emphasizing its direct influence on cardiac rhythm and highlighting hypochloremia as a potential marker of arrhythmic risk in patients with heart failure [15,16]. The key physiological and pathophysiological roles of chloride in heart failure and cardiorenal syndrome are summarized in Table 1.

## 5. Current Research on Chloride and Cardiorenal Syndrome

Multiple clinical investigations have emphasized the prognostic significance of serum chloride in heart failure (HF). Across sixteen studies, low serum chloride concentrations were associated with adverse outcomes in both hospitalized patients with acute HF (6787 individuals) and outpatients with chronic HF (18,757 individuals). Some studies also suggested a U-shaped relationship, with both hypochloremia and hyperchloremia linked to worse outcomes, although not all investigations confirmed a correlation between hyperchloremia and prognosis. In fact, several studies either omitted hyperchloremia from their analyses or found no significant association [15]. In a subset of investigations, serial chloride measurements were employed to explore whether dynamic changes in serum chloride, rather than baseline levels alone, predicted clinical outcomes. For instance, Kataoka observed an increase in serum chloride levels during episodes of worsening HF, followed by a decline after clinical stabilization [43]. Although chloride and sodium levels were often correlated, the strength of this relationship varied considerably, with some studies reporting only a modest association [12,23]. Interestingly, while hypochloremia was often linked to increased mortality, particularly in patients who also exhibited hyponatremia, the prognostic impact of chloride appeared more consistent than that of sodium. In some analyses, the association between hyponatremia and mortality weakened after adjustment for chloride levels, suggesting that chloride disturbances may play a more fundamental role [10,11,12,23,44,45]. Furthermore, in studies comparing serial changes, shifts in serum chloride concentrations tended to be more pronounced than corresponding changes in sodium [35,43,45]. A higher baseline sodium-to-chloride ratio has also been associated with a greater risk of adverse outcomes, reinforcing the emerging view of chloride as a critical cardiorenal connector [4,8]. Recent evidence increasingly points to serum chloride not merely as a passive marker, but as an active contributor to disease progression in HF, with a potentially stronger prognostic role than serum sodium across a range of heart failure syndromes [4].

## 6. How to Overcome Therapeutic Challenges in HF

Despite the established prognostic significance of chloride in HF, its potential as a therapeutic target remains unclear. Previous attempts to address hyponatremia pharmacologically have not yielded consistent improvements in clinical outcomes, which has prompted interest in targeting chloride homeostasis instead. Given chloride’s broader biological role—including its influence on neurohormonal activation and diuretic responsiveness—it is plausible that correcting chloride imbalances may confer clinical benefit [8,14].

Adjunctive use of hypertonic saline in acute decompensated heart failure has shown promise in improving diuretic responsiveness. While traditionally attributed to sodium repletion, emerging evidence suggests that the therapeutic benefit may instead be linked to chloride restoration [15,16]. Early trials demonstrated net-positive sodium balance following hypertonic saline infusion, yet patients experienced improved natriuresis and weight loss, implying a more complex mechanism, potentially mediated by chloride [46].

In contrast, sodium-restricted diets have not consistently improved outcomes in HF, and in some studies, have been associated with worse clinical trajectories [47,48]. Since dietary sodium and chloride are closely linked—primarily consumed as sodium chloride—it has been hypothesized that chloride depletion, rather than sodium excess, may be the more relevant target for intervention. However, the precise impact of dietary chloride on systemic chloride balance remains incompletely understood [14].

Sodium-free chloride supplementation, such as lysine chloride, offers a potential method to restore chloride levels without disturbing sodium balance. Early clinical observations suggest improved markers of decongestion, including enhanced natriuresis and hemoconcentration, though larger studies are needed [14,23,49].

Acetazolamide, a carbonic anhydrase inhibitor that promotes chloride retention, has also shown promise in overcoming diuretic resistance, as mentioned previously [16,50]. In the ADVOR trial, acetazolamide added to loop diuretics improved natriuresis and accelerated decongestion compared to placebo. The sustained benefit beyond the initial treatment period raises the possibility that chloride retention may influence downstream neurohormonal activity, though this remains speculative [51].

In conclusion, emerging therapies aimed at correcting chloride imbalance—whether through chloride supplementation or use of chloride-sparing diuretics—offer a novel approach to optimizing decongestion in HF. As our understanding of chloride physiology deepens, these strategies may provide important clinical benefits, particularly in patients with diuretic resistance or recurrent hypochloremia [8,14,15].

## 7. Clinical Application: Integrating Chloride Profiles into Heart Failure Management

**Figure 2:**
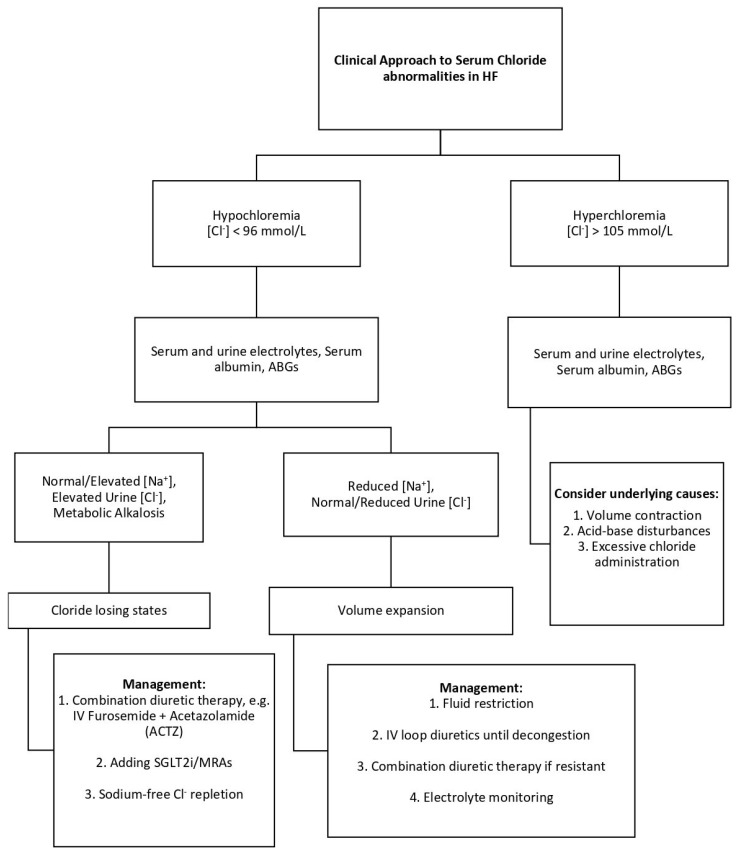
Clinical approach to serum chloride abnormalities in patients with heart failure. This algorithm outlines the evaluation and management of hypochloremia, hyperchloremia, and normal chloride levels in heart failure. Hypochloremia is further differentiated into profiles reflecting chloride depletion or hemodilution, based on sodium and urine chloride levels. Management strategies include tailored use of diuretics, fluid restriction, and chloride repletion. Hyperchloremia prompts evaluation for causes such as IV saline overload and metabolic acidosis. Serum and urine electrolytes, arterial blood gases, and volume status guide individualized therapy [15]. Abbreviations: ABGs: air blood gases; ACTZ: acetazolamide; HF: heart failure; IV: intravenous; MRAs: mineralocorticoid receptor antagonists; SGLT2i: sodium–glucose cotransporter-2 inhibitor. This is an original, clinically oriented figure developed by the authors to support practical decision-making based on chloride profiling in HF.

Recognizing chloride abnormalities provides valuable insights for guiding therapy in heart failure. For example, a patient with persistent congestion despite escalating loop diuretics, normal serum sodium, and low chloride may be experiencing true chloride depletion. In such cases, adjunctive strategies such as chloride repletion with hypertonic saline or the addition of acetazolamide may enhance diuretic responsiveness [15]. In contrast, a patient presenting with both hypochloremia and hyponatremia likely reflects water excess and hemodilution, where fluid restriction and neurohormonal modulation may be more appropriate to restore volume balance. Similarly, in patients receiving large volumes of chloride-rich intravenous fluids, close monitoring for hyperchloremia is crucial, as this may provoke renal vasoconstriction and worsen kidney function [14]. Tailoring management based on chloride profiles may help optimize decongestion and improve clinical outcomes. The clinical approach to serum chloride abnormalities in patients with heart failure is illustrated in Figure 2.

Urine chloride measurement can further refine clinical assessment. In patients with true chloride depletion, urine chloride levels are typically low, reflecting renal conservation. In contrast, patients with dilutional hypochloremia often maintain higher urine chloride concentrations, indicating that renal handling of chloride remains intact despite systemic volume overload. Although not yet standard practice, incorporating urine chloride measurements where feasible may assist in differentiating between these two clinical profiles and guide more targeted interventions—whether through chloride repletion or careful fluid management. This approach parallels the use of urine sodium levels in evaluating hyponatremia, where low urine sodium suggests volume depletion, and preserved or elevated urine sodium indicates dilutional states or salt-losing nephropathy [43,52]. Emerging evidence also suggests that urine chloride may serve as a useful marker of diuretic response and neurohormonal activation in acute heart failure, with lower uCl^−^ levels associated with worse outcomes and treatment resistance. While pilot data support its rapid bedside measurement and potential clinical utility, larger studies are needed to establish its precise role in guiding decongestive therapy [40,53,54].

## 8. Future Perspectives

Although the relationship between serum chloride levels and heart failure outcomes is increasingly supported by observational data, most available evidence derives from retrospective studies or secondary analyses of clinical trials that were not designed to investigate chloride as a primary variable. As a result, while the findings are consistent, they are limited by potential confounders and a lack of prospective validation [15]. Importantly, unlike serum sodium—which primarily reflects vasopressin (ADH) activity and water retention—serum chloride offers a broader perspective on neurohormonal activation. It is influenced not only by ADH, but also by aldosterone-driven sodium reabsorption and hydrogen ion secretion, often manifesting as metabolic alkalosis. Hypochloremia may therefore more comprehensively reflect the neurohormonal dysregulation characteristic of acute heart failure [4,55]. Furthermore, the emerging role of cardiac chloride channels in arrhythmogenesis and myocardial function lends additional biological plausibility to its prognostic relevance [15,16].

Beyond neurohormonal signaling, chloride is intricately involved in acid–base homeostasis through its effect on systemic pH. It remains unclear whether fluctuations in pH modulate the prognostic or therapeutic implications of hypochloremia, including its association with diuretic responsiveness. Future studies should seek to disentangle these complex interactions in a controlled setting [14,16].

Prospective clinical trials—particularly those investigating novel decongestive strategies such as ultrafiltration or diuretic-sparing approaches—should incorporate serum and urine chloride measurement as part of routine safety and efficacy monitoring. Systematic reporting of chloride dynamics may provide valuable mechanistic insights and help identify unintended consequences of therapeutic interventions [14,15].

Finally, with growing evidence of its prognostic value, serum chloride should be considered for inclusion in contemporary heart failure risk models. Whether its integration improves the predictive accuracy of tools that currently rely on markers such as sodium, hemoglobin, and blood urea nitrogen warrants formal investigation. If validated, this could enhance personalized risk stratification and therapeutic decision-making in heart failure management [15].

## 9. Conclusions

Chloride, long overshadowed by sodium in heart failure management, is emerging as a critical player in fluid balance, neurohormonal activation, acid–base homeostasis, and the electrical properties of the myocardial cell. Both hypochloremia and hyperchloremia are associated with adverse outcomes, with hypochloremia in particular linked to diuretic resistance, neurohormonal dysregulation, and increased mortality. The complex interplay between chloride levels and renal, cardiovascular, and hormonal mechanisms suggests that chloride is not merely a passive bystander but a dynamic regulator in the pathophysiology of heart failure and cardiorenal syndrome. As growing evidence supports its prognostic relevance, routine monitoring of serum and potentially urine chloride should be considered in clinical practice. Future studies are warranted to clarify whether therapeutic strategies targeting chloride balance—such as chloride-sparing diuretics or selective repletion—can improve patient outcomes and help overcome persistent treatment challenges in decompensated heart failure.

## Figures and Tables

**Table 1:** Key physiological and pathophysiological roles of chloride in heart failure and cardiorenal syndrome. This table summarizes the major domains influenced by chloride—volume regulation, neurohormonal activation, acid–base balance, diuretic responsiveness, and cardiac arrhythmias—along with associated mechanisms and clinically relevant details. Abbreviations: CFTR: cystic fibrosis transmembrane conductance regulator; ECF: Extracellular fluid; GI: Gastrointestinal tract; NCC: Na-Cl symporter; NKCC2: Na-K-2Cl transporter; RAAS: renin–angiotensin–aldosterone system; WNK: With-no-Kinase [K].

Chloride Mechanisms in Cardiorenal Syndrome		
Main Domain	Mechanisms/Role of Chloride	Key Details
**Volume and fluid balance**	Regulation of ECF volume Contribution to osmolarity and fluid distribution	Dilutional versus depletional hypochloremia profiles Hypochloremia linked to volume overload or diuretic use
**Neurohormonal activation**	Modulation of RAAS and sympathetic tone Sensed at macula densa—affects vasopressin and thirst pathways	Chloride deficiency stimulates renin and vasopressin release Restoration suppresses neurohormonal activation
**Acid-base regulation**	Inverse interaction with bicarbonate; influences systemic pH balance	Hyperchloremia can cause metabolic acidosis (renal or GI origin) Contribution to worse prognosis
**Diuretic responsiveness**	Regulation of WNK kinases—affects diuretic target activity and natriuretic efficiency	Hypochloremia impairs diuretic efficiency via WNK activation (upregulation of NCC and NKCC2 → increased sodium reabsorption)
**Cardiac arrhythmias**	Membrane potential control, repolarization, and myocyte volume	CFTR downregulation in HF linked to arrhythmias and hypertrophy Intracellular pH and potassium homeostasis

## Data Availability

No new data were created or analyzed in this study. Data sharing is not applicable to this article.

## Abbreviations

The following abbreviations are used in this manuscript:

ACTZ	Acetazolamide
ADH	Antidiuretic hormone/vasopressin
ADVOR	Acetazolamide in Decompensated Heart Failure with Volume Overload Trial
ATP	Adenosine Triphosphate
BEST	Beta-Blocker Evaluation of Survival Trial
BNP	B-type natriuretic peptide
Cl	Chloride
CRS	Cardiorenal syndrome
CTFR	Cystic fibrosis transmembrane conductance regulator
GFR	Glomerular filtration rate
ECF	Extracellular fluid
GIT	Gastrointestinal tract
HCO3	Bicarbonate
HF	Heart failure
IV	Intravenous
K	Potassium
MRAs	Mineralocorticoid receptor antagonists
Na	Sodium
NCC	Na^+^-Cl^−^ symporter
NHE	Na^+^-H^+^ exchanger
NKCC2	Na^+^-K^+^-2Cl^−^ transporter
RAAS	Renin–angiotensin–aldosterone system
ROSE	Renal Optimization Strategies Evaluation
RTA	Renal tubular acidosis
SGLT2i	Sodium–glucose cotransporters-2 inhibitors
TOPCAT	Treatment of Preserved Cardiac Function Heart Failure with an Aldosterone Antagonist Trial
uCl^−^	Urine chloride
uNa^+^	Urine sodium
VICTORIA	Vericiguat Global Study in Subjects with Heart Failure with Reduced Ejection Fraction
WNK	With-no-Lysine [K]

## References

[1] Rangaswami J., Bhalla V., Blair J.E.A., Chang T.I., Costa S., Lentine K.L., Lerma E.V., Mezue K., Molitch M., Mullens W. (2019). Cardiorenal Syndrome: Classification, Pathophysiology, Diagnosis, and Treatment Strategies: A Scientific Statement from the American Heart Association. Circulation.

[2] Dutta A., Saha S., Bahl A., Mittal A., Basak T. (2023). A comprehensive review of acute cardio-renal syndrome: Need for novel biomarkers. Front. Pharmacol..

[3] Mitsas A.C., Elzawawi M., Mavrogeni S., Boekels M., Khan A., Eldawy M., Stamatakis I., Kouris D., Daboul B., Gunkel O. (2022). Heart Failure and Cardiorenal Syndrome: A Narrative Review on Pathophysiology, Diagnostic and Therapeutic Regimens—From a Cardiologist’s View. J. Clin. Med..

[4] Kazory A., Costanzo M.R. (2020). The dynamic relationship between serum chloride and cardiorenal syndrome. Rev. Cardiovasc. Med..

[5] Doukky R., Avery E., Mangla A., Collado F.M., Ibrahim Z., Poulin M.-F., Richardson D., Powell L.H. (2016). Impact of Dietary Sodium Restriction on Heart Failure Outcomes. JACC Heart Fail..

[6] Mazón-Ruiz J., Romero-González G., Sánchez E., Banegas-Deras E.J., Salgado-Barquinero M., la Varga L.G., Bande-Fernández J.J., Gorostidi M., Alcázar R. (2024). Hypertonic saline and heart failure: “sodium-centric” or “chlorine-centric”?. Nefrologia.

[7] Griffin M., Soufer A., Goljo E., Colna M., Rao V.S., Jeon S., Raghavendra P., D’aMbrosi J., Riello R., Coca S.G. (2020). Real World Use of Hypertonic Saline in Refractory Acute Decompensated Heart Failure: A U.S. Center’s Experience. JACC Heart Fail..

[8] Kazory A., Ronco C. (2020). Emergence of Chloride as an Overlooked Cardiorenal Connector in Heart Failure. Blood Purif..

[9] O’Connor C.M., Ahmad T. (2015). The Role of Sodium and Chloride in Heart Failure: Does It Take Two to Tango?. J. Am. Coll. Cardiol..

[10] Grodin J.L., Simon J., Hachamovitch R., Wu Y., Jackson G., Halkar M., Starling R.C., Testani J.M., Tang W.W. (2015). Prognostic Role of Serum Chloride Levels in Acute Decompensated Heart Failure. J. Am. Coll. Cardiol..

[11] Grodin J.L., Verbrugge F.H., Ellis S.G., Mullens W., Testani J.M., Wilson Tang M.D.W.H. (2016). The Importance of Abnormal Chloride Homeostasis in Stable Chronic Heart Failure. Circ. Heart Fail..

[12] Testani J.M., Hanberg J.S., Arroyo J.P., Brisco M.A., ter Maaten J.M., Wilson F.P., Bellumkonda L., Jacoby D., Tang W.W., Parikh C.R. (2016). Hypochloraemia is strongly and independently associated with mortality in patients with chronic heart failure. Eur. J. Heart Fail..

[13] Tan Z., Liu Y., Hong K. (2024). The association between serum chloride and mortality in ICU patients with heart failure: The impact of bicarbonate. Int. J. Cardiol..

[14] Arora N. (2023). Serum Chloride and Heart Failure. Kidney Med..

[15] Zandijk A.J.L., van Norel M.R., Julius F.E.C., Sepehrvand N., Pannu N., McAlister F.A., Voors A.A., Ezekowitz J.A. (2021). Chloride in Heart Failure. JACC Heart Fail..

[16] Cuthbert J.J., Bhandari S., Clark A.L. (2020). Hypochloraemia in Patients with Heart Failure: Causes and Consequences. Cardiol. Ther..

[17] Van den Eynde J., Martens P., Dauw J., Nijst P., Meekers E., ter Maaten J.M., Damman K., Filippatos G., Lassus J., Mebazaa A. (2024). Serum Chloride and the Response to Acetazolamide in Patients with Acute Heart Failure and Volume Overload: A Post Hoc Analysis from the ADVOR Trial. Circ. Heart Fail..

[18] Grodin J.L., Testani J.M., Pandey A., Sambandam K., Drazner M.H., Fang J.C., Tang W.W. (2018). Perturbations in serum chloride homeostasis in heart failure with preserved ejection fraction: Insights from TOPCAT. Eur. J. Heart Fail..

[19] Mentz R.J., Mulder H., Mosterd A., Sweitzer N.K., Senni M., Butler J., Ezekowitz J.A., Lam C.S., Pieske B., Ponikowski P. (2021). Clinical Outcome Predictions for the VerICiguaT Global Study in Subjects with Heart Failure with Reduced Ejection Fraction (VICTORIA) Trial: VICTORIA Outcomes Model. J. Card. Fail..

[20] Maryam Varghese T.P., Tazneem B. (2024). Unraveling the complex pathophysiology of heart failure: Insights into the role of renin-angiotensin-aldosterone system (RAAS) and sympathetic nervous system (SNS). Curr Probl Cardiol.

[21] Sayer G., Bhat G. (2014). The Renin-Angiotensin-Aldosterone System and Heart Failure. Cardiol. Clin..

[22] Bankir L., Bichet D.G., Morgenthaler N.G. (2017). Vasopressin: Physiology, assessment and osmosensation. J. Intern. Med..

[23] Hanberg J.S., Rao V., Ter Maaten J.M., Laur O., Brisco M.A., Perry Wilson F., Grodin J.L., Assefa M., Broughton J.S., Planavsky N.J. (2016). Hypochloremia and Diuretic Resistance in Heart Failure: Mechanistic Insights. Circ. Heart Fail..

[24] Ward A., Heel R.C. (1984). Bumetanide. A review of its pharmacodynamic and pharmacokinetic properties and therapeutic use. Drugs.

[25] Rivera F.B., Alfonso P., Golbin J.M., Lo K., Lerma E., Volgman A.S., Kazory A. (2021). The Role of Serum Chloride in Acute and Chronic Heart Failure: A Narrative Review. Cardiorenal Med..

[26] Radulović B., Potočnjak I., Terešak S.D., Trbušić M., Vrkić N., Malogorski D., Starčević N., Milošević M., Frank S., Degoricija V. (2016). Hypochloraemia as a predictor of developing hyponatraemia and poor outcome in acute heart failure patients. Int. J. Cardiol..

[27] Kataoka H. (2021). Chloride in Heart Failure Syndrome: Its Pathophysiologic Role and Therapeutic Implication. Cardiol. Ther..

[28] Palmer B.F., Clegg D.J. (2019). Hyperchloremic normal gap metabolic acidosis. Minerva Endocrinol..

[29] Sharma S., Hashmi M.F., Aggarwal S. (2025). Hyperchloremic Acidosis. StatPearls.

[30] Kotchen T.A., Luke R.G., Ott C.E., Galla J.H., Whitescarver S. (1983). Effect of chloride on renin and blood pressure responses to sodium chloride. Ann. Intern. Med..

[31] Kirchner K.A., Kotchen T.A., Galla J.H., Luke R.G. (1978). Importance of chloride for acute inhibition of renin by sodium chloride. Am. J. Physiol..

[32] Kataoka H. (2017). The “chloride theory”, a unifying hypothesis for renal handling and body fluid distribution in heart failure pathophysiology. Med. Hypotheses.

[33] Wilcox C.S., Testani J.M., Pitt B. (2020). Pathophysiology of Diuretic Resistance and Its Implications for the Management of Chronic Heart Failure. Hypertension.

[34] Testani J.M., Brisco M.A., Turner J.M., Spatz E.S., Bellumkonda L., Parikh C.R., Tang W.W. (2014). Loop diuretic efficiency: A metric of diuretic responsiveness with prognostic importance in acute decompensated heart failure. Circ. Heart Fail..

[35] Ter Maaten J.M., Damman K., Hanberg J.S., Givertz M.M., Metra M., O’Connor C.M., Teerlink J.R., Ponikowski P., Cot-ter G., Davison B. (2016). Hypochloremia, Diuretic Resistance, and Outcome in Patients with Acute Heart Failure. Circ. Heart Fail..

[36] Grodin J.L., Sun J.-L., Anstrom K.J., Chen H.H., Starling R.C., Testani J.M., Tang W.W. (2017). Implications of Serum Chloride Homeostasis in Acute Heart Failure (from ROSE-AHF). Am. J. Cardiol..

[37] Hoorn E.J., Ellison D.H. (2012). WNK kinases and the kidney. Exp. Cell Res..

[38] Koulouridis I., Koulouridis E. (2023). The Integral Role of Chloride & with-No-Lysine Kinases in Cell Volume Regulation & Hypertension. Int. J. Nephrol. Renov. Dis..

[39] Lameire N. (2023). Renal Mechanisms of Diuretic Resistance in Congestive Heart Failure. Kidney Dial..

[40] Guzik M., Zymliński R., Ponikowski P., Biegus J. (2025). Urine chloride trajectory and relationship with diuretic response in acute heart failure. ESC Heart Fail..

[41] Adkins G.B., Curtis M.J. (2015). Potential role of cardiac chloride channels and transporters as novel therapeutic targets. Pharmacol. Ther..

[42] Vaughan-Jones R.D., Spitzer K.W., Swietach P. (2009). Intracellular pH regulation in heart. J. Mol. Cell Cardiol..

[43] Kataoka H. (2018). Dynamic changes in serum chloride concentrations during worsening of heart failure and its recovery following conventional diuretic therapy: A single-center study. Health Sci. Rep..

[44] Cuthbert J.J., Pellicori P., Rigby A., Pan D., Kazmi S., Shah P., Clark A.L. (2018). Low serum chloride in patients with chronic heart failure: Clinical associations and prognostic significance. Eur. J. Heart Fail..

[45] Kondo T., Yamada T., Tamaki S., Morita T., Furukawa Y., Iwasaki Y., Kawasaki M., Kikuchi A., Ozaki T., Sato Y. (2018). Serial Change in Serum Chloride During Hospitalization Could Predict Heart Failure Death in Acute Decompensated Heart Failure Patients. Circ. J. Off. J. Jpn. Circ. Soc..

[46] Gandhi S., Mosleh W., Myers R.B.H. (2014). Hypertonic saline with furosemide for the treatment of acute congestive heart failure: A systematic review and meta-analysis. Int. J. Cardiol..

[47] Mahtani K.R., Heneghan C., Onakpoya I., Tierney S., Aronson J.K., Roberts N., Hobbs F.D.R., Nunan D. (2018). Reduced Salt Intake for Heart Failure: A Systematic Review. JAMA Intern. Med..

[48] Ezekowitz J.A., Colin-Ramirez E., Ross H., Escobedo J., Macdonald P., Troughton R., Saldarriaga C., Alemayehu W., McAlister F.A., Arcand J. (2022). Reduction of dietary sodium to less than 100 mmol in heart failure (SODIUM-HF): An international, open-label, randomised, controlled trial. Lancet Lond. Engl..

[49] Ter Maaten J.M., Damman K. (2018). Chloride, what else?. Eur. J. Heart Fail..

[50] Verbrugge F.H., Martens P., Ameloot K., Haemels V., Penders J., Dupont M., Tang W.H.W., Droogné W., Mullens W. (2019). Acetazolamide to increase natriuresis in congestive heart failure at high risk for diuretic resistance. Eur. J. Heart Fail..

[51] Mullens W., Dauw J., Martens P., Verbrugge F.H., Nijst P., Meekers E., Tartaglia K., Chenot F., Moubayed S., Dierckx R. (2022). Acetazolamide in Acute Decompensated Heart Failure with Volume Overload. N. Engl. J. Med..

[52] Verbrugge F.H., Steels P., Grieten L., Nijst P., Tang W.H.W., Mullens W. (2015). Hyponatremia in Acute Decompensated Heart Failure: Depletion Versus Dilution. J. Am. Coll. Cardiol..

[53] Shah V., Cordwin D., Hummel S.L., Dorsch M.P. (2025). Chloride dipstick to rapidly estimate urine sodium during diuresis in acute heart failure. Pharmacother. J. Hum. Pharmacol. Drug Ther..

[54] Nawrocka-Millward S., Biegus J., Fudim M., Guzik M., Iwanek G., Ponikowski P., Zymliński R. (2024). The role of urine chloride in acute heart failure. Sci. Rep..

[55] Kazory A., Ronco C., Koratala A. (2024). Cardiorenal Interactions in Acute Heart Failure: Renal Proximal Tubules in the Spotlight. Cardiorenal Med..

